# The BabySaver: Design of a New Device for Neonatal Resuscitation at Birth with Intact Placental Circulation

**DOI:** 10.3390/children8060526

**Published:** 2021-06-21

**Authors:** James Ditai, Aisling Barry, Kathy Burgoine, Anthony K. Mbonye, Julius N. Wandabwa, Peter Watt, Andrew D. Weeks

**Affiliations:** 1 Sanyu Africa Research Institute, Mbale Regional Referral Hospital, Pallisa Road, Mbale P.O. Box 2190, Uganda; 2 Sanyu Research Unit, Department of Women’s and Children’s Health, University of Liverpool, Liverpool Women’s Hospital, Crown Street, Liverpool L8 7SS, UK; aweeks@liv.ac.uk; 3 Faculty of Health Sciences, Busitema University, Pallisa Road, Mbale P.O. Box 2190, Uganda; gjwandabwa@yahoo.com; 4 The Department of Medical Engineering and Physics, Kings College London, London SE5 9RS, UK; aislingfbarry@gmail.com; 5 Neonatal Unit, Mbale Regional Referral Hospital, Pallisa Road, Mbale P.O. Box 2190, Uganda; Kathy.burgoine@liv.ac.uk; 6 School of Public Health, Makerere University College of Health Sciences, Kampala P.O. Box 7072, Uganda; akmbonye@yahoo.com; 7 Department of Medical Physics and Clinical Engineering, University of Liverpool, Liverpool L7 8XP, UK; rjpwatt@gmail.com

**Keywords:** intact cord, resuscitation, placental circulation, design, BabySaver

## Abstract

The initial bedside care of premature babies with an intact cord has been shown to reduce mortality; there is evidence that resuscitation of term babies with an intact cord may also improve outcomes. This process has been facilitated by the development of bedside resuscitation surfaces. These new devices are unaffordable, however, in most of sub-Saharan Africa, where 42% of the world’s 2.4 million annual newborn deaths occur. This paper describes the rationale and design of BabySaver, an innovative low-cost mobile resuscitation unit, which was developed iteratively over five years in a collaboration between the Sanyu Africa Research Institute (SAfRI) in Uganda and the University of Liverpool in the UK. The final BabySaver design comprises two compartments; a tray to provide a firm resuscitation surface, and a base to store resuscitation equipment. The design was formed while considering contextual factors, using the views of individual women from the community served by the local hospitals, medical staff, and skilled birth attendants in both Uganda and the UK.

## 1. Introduction

Sub-Saharan Africa is reported to have 1 million newborn deaths annually, accounting for 42% of the world’s total newborn deaths [1,2,3]. The need for any form of resuscitation at birth is 10% globally [4] but rates are higher in sub-Saharan African countries with reported rates of 24–32% [5,6,7]. Most babies needing resuscitation require only simple stimulation (drying and rubbing), which can be performed at the mother’s side without transferring the baby to another resuscitation surface; 3–6% of all babies (approximately 6 million/year) require further (basic) neonatal resuscitation, comprising stimulation plus bag and mask ventilation; very few need advanced resuscitation (chest compression, endotracheal intubation, and medication) [4]. It is estimated that the provision of universal access to basic resuscitation of newborns could save 904,000 newborn lives annually, with additional reductions in chronic neurological abnormalities [8]. Historically, however, the focus has been on staff training and the provision of resuscitation areas away from the mother.

Providing neonatal resuscitation at the mother’s bedside with an intact umbilical cord is potentially a high benefit practice with major global benefits; it enables physiological benefits for the baby, keeps the midwife with the mother in the vital few minutes after birth, and allows the mother to stay with her newborn, preventing any suspicion of malpractice [9,10,11].

In the United Kingdom, Hutchon and colleagues explored ways of achieving resuscitation with an intact cord. This resulted in the development of a small mobile bedside resuscitation trolley, later commercialised as the LifeStart trolley (Inspiration Healthcare, Crawley, UK) [12,13,14,15]. This was followed in the Netherlands by Concord: a purpose-built resuscitation table for physiological-based cord clamping in preterms [16,17,18,19]. Other devices developed to date include the NOOMA cart in the USA and the INSPiRE trolley in Canada [20]. However, these innovations are not possible in low-resource settings as they require expensive equipment, a hospital base, and mains electricity.

The BabySaver is a simple mobile vacuum-moulded, oval plastic assembly resuscitation unit developed by a team of designers at the Royal Liverpool University Hospital, researchers at SAfRI, and clinicians at Liverpool Women’s Hospital (LWH) and Mbale Regional Referral Hospital. It is the first medical device designed to promote neonatal resuscitation with an intact cord in low-resource settings. The final device prototype has since undergone phase I and II clinical testing studies in Uganda, reported elsewhere.

This paper discusses the rationale and design of a medical device, including the nature and effect of contextual factors on its final design.

### The Rationale for the BabySaver Newborn Resuscitation Device

The primary objective for developing the BabySaver device was to reduce intrapartum-related deaths in low-resource delivery environments. We aimed to develop a device that:

- provides a stable, flat but firm surface for the baby [10] and enables the skilled birth attendant to resuscitate during the first “golden minute” after birth [21]
- facilitates resuscitation of depressed neonates at birth whilst keeping the umbilical cord intact [9,10,11]
- allows resuscitation of the babies by the mother’s side [9,10,11]
- prevents separation of the skilled birth attendant and mother, so that the care of the mother is not interrupted
- allows the mother to remain with the baby during its initial support, instead of removing it from her sight during these vital moments
- facilitates newborn resuscitation when only one skilled birth attendant is present.

The BabySaver newborn resuscitation device was designed from simple plastics to maximise sustainability in Uganda’s public health facilities and replicability in low-resource settings across the world.

## 2. Materials and Methods

### 2.1. Team

The design and development of the BabySaver device were coordinated by a small team of academics working within the Sanyu Research Unit, the Royal Liverpool University Hospital Department of Physics and Engineering, in collaboration with SAfRI. SAfRI is a not-for-profit non-government organisation, based at Mbale Regional Referral Hospital in Eastern Uganda; it and the Sanyu Research Unit in Liverpool were set up to research low-cost innovations and improve the care of mothers and their newborns. The team includes those who were responsible for the development of a high-end bedside resuscitation trolley for the US and European market, sold as the “Lifestart” trolley [15].

### 2.2. Design Process

**Figure 1:**
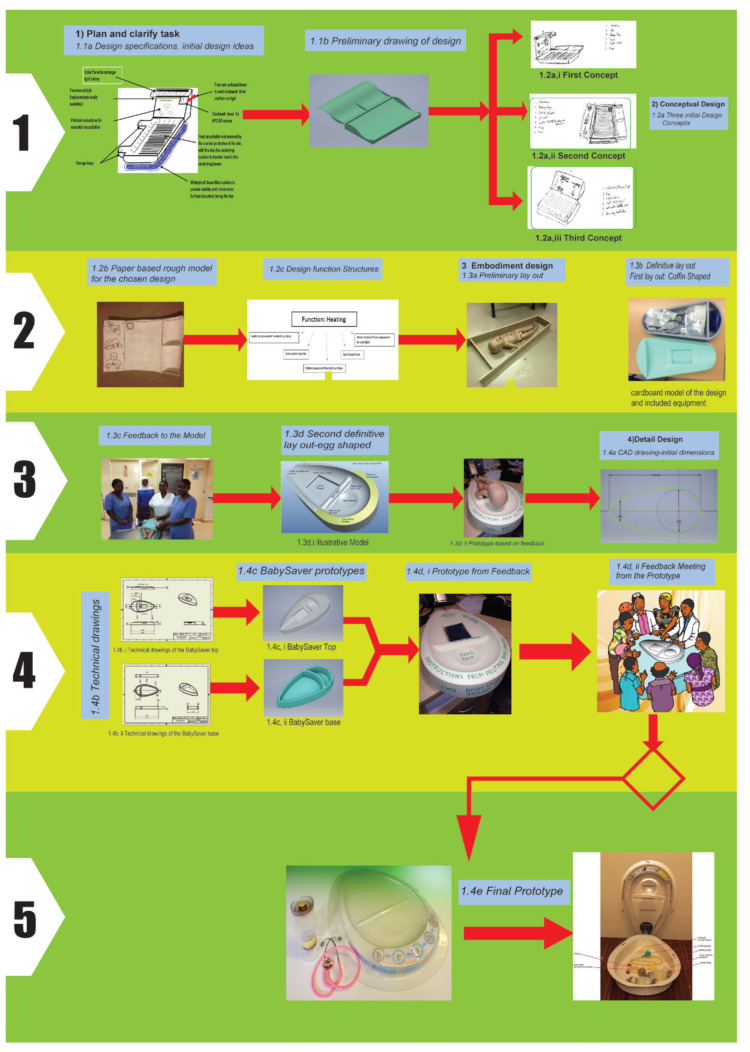
Steps in the planning and design process. (**1**). First phase, plan and clarify the task. (**2**). Second phase, the conceptual design. (**3**). Third phase, embodiment design. (**4**). Fourth phase, detailed design. (**5**). Outcome from all phases.

The BabySaver underwent systematic design using the framework of engineering design [22]. The design took place in four main phases: (1) plan and clarify the task, (2) the conceptual design, (3) embodiment design, and (4) detailed design. Figure 1 shows a summary of the design process specific to the BabySaver based on this framework [22].

### 2.3. Plan and Clarify the Task

The development of the device was informed by the best practice reviews that recommend delayed cord clamping at birth [23,24,25], the experience of using the LifeStart trolley in the United Kingdom [14,15], and recommendations for promoting delayed cord clamping for transition at birth [26,27,28].

Though local guidelines in Uganda still recommend immediate cord clamping [29], many skilled birth attendants practice a degree of delayed cord clamping; an audit at Mbale regional referral hospital in 2016 found a median time to cord clamping of 87 s in vaginal births [30]. This meant that delayed cord clamping should not be difficult to implement if there could be a device to facilitate resuscitation at the mother’s side.

#### 2.3.1. Design Team Formation

In February 2015, a design team was formed, composed of Chris Dewhurst (consultant neonatologist), Julius Wandabwa (professor of obstetrics), Peter Watt (design engineer), James Ditai (research fellow), Julian Abeso (paediatrician), Bill Yoxall (consultant neonatologist), Sam Ononge (consultant obstetrician), Lelia Duley (professor of clinical trials) and Andrew Weeks (professor of international maternal health). A face-to-face meeting was held in the department of women’s and children’s health with some members of the design team, whilst others contributed virtually or through emailed comments. The meetings discussed resuscitation and cord clamping at birth in the delivery rooms of Uganda, design idea, initial design features, requirements, and constraints.

The design team proposed design specifications at this stage based on their experience, observation of the delivery environment, and informal consultation with staff of Mbale regional referral hospital in Uganda and LWH in the United Kingdom. Peter Watt and Aisling Barry, an MSc Engineering student, worked on the design process with further input from Nick Bettles (Inspiration Healthcare, Crawley, UK), Tony Fisher (professor and head of clinical engineering at Royal Liverpool University Hospital), Kathy Burgoine (neonatologist in Mbale), and Dot Lambert (research coordinator for the Sanyu Research Unit, University of Liverpool). Figure 1(1.1a) shows the initial design features. This resulted in an initial design that was used to seek funding.

#### 2.3.2. Other Stakeholders

The design team enlisted the assistance of stakeholders who might interact with the device at different design phases in Uganda and the United Kingdom. These included end-users (women and their attendants, students of nursing, midwifery, and medicine, interns, a cleaner, midwives, nurses, medical officers, paediatricians, and obstetricians), developers (design and production engineers), regulatory authorities (the National Drugs Authority in Uganda, the Department of Medical Devices at the Ugandan Ministry of Health, and the Uganda National Council for Science and Technology), policymakers (the Ugandan Ministry of Health and the World Health Organization), and funders (Sir Halley Stewart, Grand Challenges Canada).

#### 2.3.3. Problem Identification and Design Specification

The problems with the current method of resuscitation at birth were established through the personal experiences of the design team, ongoing consultation with relevant stakeholders, and observation of the delivery room facilities of Mulago National Referral Hospital and Mbale Regional Referral Hospital. Table 1 shows the resuscitation situational analysis carried out for Ugandan delivery environments. We modified the initial design specification to include what was desired from the new design (Table 2). We revised the design in line with the revised specification to produce the preliminary drawing in Figure 1(1.1b). A £14,300 funding proposal for the development of the modified design was submitted to the Sir Halley Stewart Trust and granted in October 2015.

### 2.4. Conceptual Design

#### 2.4.1. Initial Design Concepts

The first concepts had a slatted surface to place the baby on, a solar-powered light, and a mechanical timer. The design was to be stored in the sun when not in use, and the resulting heat was stored in a solar-heated thermal capacitor (Figure 1(1.2a,i)).

The second concept was a box design with hinged flaps for storage and instructions, and a slot for a reusable heat gel pack to be inserted under the surface to diffuse heat throughout the design.

The heat gel pack was in a liquid state when inactive and solid form when active. The gel pack contained sodium acetate and water, activated by pressure on the inside metallic chip to generate heat through exothermic reaction to the design and inactivated under direct sunlight or boiling in the autoclave. The lights down each side of the design were for use at night or during maternity unit power cuts. They were intended to be powered by rechargeable batteries. The design folded easily for transport (Figure 1(1.2a,ii)).

In the third concept, a hot water bottle would be used for heat generation, and the heat stored in a bean bag base, which allowed the design to be placed on uneven surfaces, such as the mother’s abdomen or legs. The lid functioned as both an equipment store and as a place to display the instructions (Figure 1(1.2a,iii)).

##### Choice of a Design Concept

Meetings were held with several stakeholders who evaluated the three designs and chose the second design. Their choice was based on its ease of use, replacement of parts, and infection control. The chosen design was seen as minimizing the space needed for resuscitation equipment and did not interfere with the practice of resuscitation. The heat gel pack was considered easy to maintain and could ensure the constant availability of the device. The solar-powered design would need to be always taken out for sunshine charging.

##### Heat Gel Pack

The feasibility of using a sunlight-activated heat gel pack for heat generation was tested in simple experiments. The used (solid) gel pack was exposed to the sunshine in Uganda to assess the time and ambient temperature required to melt it. Three hours of sun exposure at a maximum air temperature of 26.2 °C caused partial melting of the gel, but not to state where it could be reactivated. A second used gel pack was boiled in an autoclave at 103 °C for less than 5 min, wrapped in a linen cloth to prevent melting of the plastic shell of the gel pack against the metal. This led to the complete melting of the crystals.

Finally, questions about the use of the gel pack were raised in subsequent feedback meetings. Though both boiling and autoclaving could regenerate the gel pack, some users wanted to know if just pouring boiling water from a kettle over the pack would work instead, due to the common availability of kettles in the delivery suites. This indeed would melt the pack by direct boiling of the pack in the kettle with water.

##### Methods for Design Production

We explored two methods to produce the design; injection moulding (which was expensive and rejected) and thermoforming. Thermoforming is cheap, achieves less complicated shapes, and was subsequently chosen for mass production of the finished design [22].

##### Design Materials and Choices

Materials that were suitable for thermoforming were compared with the requirements for the design. The design needed to be made of a material that is strong, heat resistant, and that does not degrade when treated with bleach. The material of the device also needed to be biologically compatible. Consultation with a local plastics company ended in the recommendation to use Polyethylene terephthalate glycol-modified (PETG). PETG is a copolymerization of PET, which is a semicrystalline plastic. The addition of glycol prevents crystallization and lowers the melting temperature of the plastic.

#### 2.4.2. Paper-Based Rough Design Model

PW made the first rough model out of hard paper and glue for the chosen design concept in 2015 (Figure 1(1.2b)).

#### 2.4.3. Design Function Structures

Design solutions were generated with corresponding diagrams for each proposed device function or specification, using the brainstorming method. Figure 1(1.2c) shows an example of the design solutions and a diagram drawn for the heating function of the design. Other functions included storage, instruction display, light, and choice of materials.

##### Choice of Helping Babies Breathe (HBB) Instructions

The resuscitation instructions to be displayed on the design were chosen with the input of midwife Chiara Mosley, a neonatal resuscitation trainer from LWH. Initially, four stages of resuscitation had been recommended for displaying on the device, but later we choose to illustrate the key steps according to the HBB algorithm [31].

We initially planned to obtain permission from the HBB program to use their drawings as a pictorial illustration of instructions, but later produced our own. Two medical students (Bethany Harrison and Nathan Thompson) on elective placement designed the initial pictorial instructions, which were subsequently revised.

### 2.5. Embodiment Design

The stakeholders agreed with the purpose, content, scope, dimensions, and function of the design.

#### 2.5.1. Preliminary Layout

##### Feedback Mbale Midwives

We sought feedback about the paper-based rough model from midwives in Mbale via group discussions in April 2015. The feedback included:

(i) The rough model was not long enough for the baby and changes to the dimensions were proposed.
(ii) The design required neck support as an add-in, to promote a natural neutral position during resuscitation.
(iii) There were concerns that the timer could not sustain the bleaching effects of Sodium hypochlorite (Jik) solution following prolonged and frequent cleaning.
(iv) They agreed with the need to include the storage tray for the pieces of equipment for resuscitation.
(v) They proposed to add a valley on the top surface of the design as a slot for the gel pack. This would allow easier inserting and cleaning of the gel pack.
(vi) Incorporating a light source in the tray would be incompatible with several specifications, especially “low cost and easy to sterilise using local methods”.
(vii) Light and timer were henceforth decided to be removed from the list and made as separate components to store in the design when needed.

##### Commonwealth and FIGO Fellows

We presented the modified design, alongside the rough design model, to the Commonwealth and FIGO Wellbeing Fellows (Fred Bisso, consultant ENT surgeon; Julian Abeso, paediatrician; Julius Wandabwa, obstetrician) in a meeting at the University Liverpool. They agreed with the proposed changes, one participant emphasizing that “even if the light and timer were separate or away for repair, the current design device could still be used”. However, there was an argument against the neck support due to the different sizes of the baby and hence the need for different sizes of neck supports.

##### Positions of the Device at the Time of Resuscitation

Initially, the design was planned to be used on either the mother’s abdomen or delivery bed. Stakeholders were concerned about the position of the maternal abdomen for the design at the time of resuscitation due to its weight and that of equipment. Further, the position of the abdomen and the side of the mother would not allow efficient blood transfer to the newborn by gravity. However, positioning the design in between the mother’s legs on the delivery bed was considered appropriate to allow placental circulation with the umbilical cord intact. This generated an add-on design specification that ensures the baby is as close as possible to the mother. The design further had to assume a shape that fits in between the mother’s legs in the lithotomy position. This formed the design for the preliminary layout.

##### Assembly of the Preliminary Layout

We took random measurements of the differently sized abdomen of gravid women and took the largest length and width for the design. Four pieces of timber were assembled to construct the design with narrow and broad ends. Figure 1(1.3a) shows the design in its preliminary layout with a model of the baby.

##### Cardboard Drawings

The team created a cardboard model showing the curve and dimensions of the preliminary layout, which was presented to midwives and obstetricians via face-to-face and international meetings. In these meetings, we sought feedback about the design and the process of use in a simulated labour environment from a lone midwife to a hospital team.

The feedback about the model in practical use helped identify the main areas of the design for remodelling.

#### 2.5.2. First Version of Definitive Layout

We constructed a prototype following approval of the preliminary layout and materials. This was not a fully functional prototype, but a scale model made to demonstrate the functions and dimensions of the finished design.

The model was made from cardboard. Figure 1(1.3b) shows the design with the suggested contents. The sloped semicircle is where the picture of the resuscitation instructions would go.

##### Feedback on the First Version of Definitive Layout

The prototype was presented to 37 end users of Mulago national referral hospital and Mbale regional referral hospital, and two health centres in December 2015. These viewed the design as a device that could provide an additional location to resuscitate the baby without taking the midwife away from the delivery suite. However, the negative responses included the design’s resemblance to a coffin, the unlikelihood of it fitting onto current beds, and dissatisfaction with current resuscitation practices. They recommended making the design’s shape friendlier (less coffin-like), broadening one end and narrowing the curve at the other end. All agreed to the need for neck support but differed in opinion on its height. They proposed excluding the timer and providing multiple trays or coverings for sterile purposes. Figure 1(1.3c) shows the midwives in Mbale with the prototype in December 2016.

They suggested a clear tray to allow visibility of the pieces of equipment for resuscitation inside, child-friendly stickers, and more curves to the design. Several people requested that the tray be more ergonomically designed for resuscitation with a bag and mask, mostly centred on the rounding of the sharp edge where the curved section of the tray meets the instruction section. They also proposed making the base of the design flatter to look more like the weighing scales which are widely used. This was seen to have the added benefit of providing more room for the babies’ shoulders.

##### Feedback on the Contents of the BabySaver Design

We sought feedback on the desired contents in the design. While most users were satisfied with the proposed auxiliary components, some wanted additional equipment. This ranged from thermometers to caps for babies, drugs, and cannulas. Though most people interviewed desired a timer to be included in the tray, they were happy with the current use of a wall clock for time.

However, it was subsequently argued that additional components would take focus away from the main key steps of resuscitation. Further, including consumables in the design would discourage use when they were gone or out of stock. We hence agreed to keep the main contents for resuscitation in the design. We hence classified contents into the design as essential and optional (Table 3).

#### 2.5.3. Second Version of Definitive Layout

The definitive layout was proposed by Peter Watt and developed as part of the MSc engineering project for Aisling Barry. To escape the coffin shape, an egg shape was proposed, designed with more complex intersecting curves using Pro/Engineer 3D solid modelling software (Figure 1(1.3d,i)); and produced the first model using a computer numerical control (CNC) router in rigid modelling foam (Figure 1(1.3d,ii)).

##### Feedback on the Second Version of Definitive Layout

The egg-shaped 3D routed prototype in April 2017 was reviewed by paediatricians, obstetricians, doctors, and midwives in Uganda who all completed feedback sheets to comment on its design, shape, and functionality. This prototype was also reviewed by staff at the LWH neonatal intensive care unit (NICU), who provided written feedback. The final layout was achieved by December 2017.

### 2.6. Detailed Design

We produced the technical drawings of the version of the design to be made into the first functional prototype.

#### 2.6.1. CAD Drawings

The revised design was drawn up using Autodesk Inventor, a computer-aided design software package. Figure 1(1.4a) shows a sample of the technical drawings of the final design.

#### 2.6.2. Technical Drawings

Figure 1(1.4b) shows the sample technical drawings of the tray (i) and the base (ii). The drawings were done in a third-angle projection to ISO standards. All designs are intended for use with a 2 mm thick initial sheet of PET-G.

#### 2.6.3. Prototype Initial Shape

Both the tray (Figure 1(1.4c,i)) and base (Figure 1(1.4c,ii)) for the initial shape of the design were constructed independently. The supporting walls of the base were hollow and set at a wide draft angle of 5 degrees to work better with the draw ratio constraints of thermoforming.

The top tray was constructed to fit snugly over the base, with 20 mm in between to provide passage for the users’ fingers when lifting the top tray. The addition of the neck support and the increased smoothness of transitions between surfaces can be seen in the revised version of the design.

#### 2.6.4. Feedback from Users in Uganda

We sought feedback in an interactive manner on the most recent version of the device (Figure 1(1.4d,i)) from women, health workers, and policymakers in Mbale regional referral hospital, Mulago national referral hospital, and the Ministry of Health. The following major changes were subsequently recommended.

The use of the gel packs was potentially troublesome; they could not be recharged rapidly or easily, there was a high chance of theft or damage, and there was a relatively high cost of replacement. We decided, therefore, to remove the gel packs, and hence a gel pack recess from the design in April 2017. This is shown in the final prototype manufactured for use in clinical testing, Figure 1(1.4e).

## 3. Results

### 3.1. Device Class

The device is a class I medical device [32] due to its transient, non-invasive, active therapeutic nature [33] and minimal risks established during the risk assessment for resuscitation [34] and COVID-19 [35]. Table 4 shows the risk assessment for the design.

### 3.2. Device Description

The BabySaver is a simple, mobile, vacuum-moulded, and oval plastic assembly specifically designed to be used between the mother’s legs or by the mother’s side on the delivery bed. It provides a firm flat platform to resuscitate a depressed neonate at birth while the umbilical cord remains intact.

**Figure 2:**
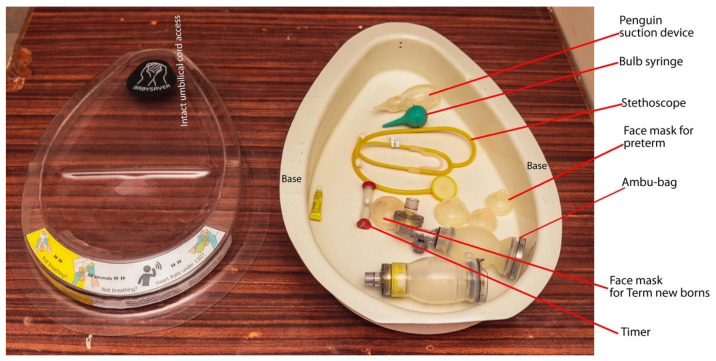
BabySaver design (current prototype). The BabySaver comprises two compartments: a tray at the top and a base at the bottom.

**Figure 3:**
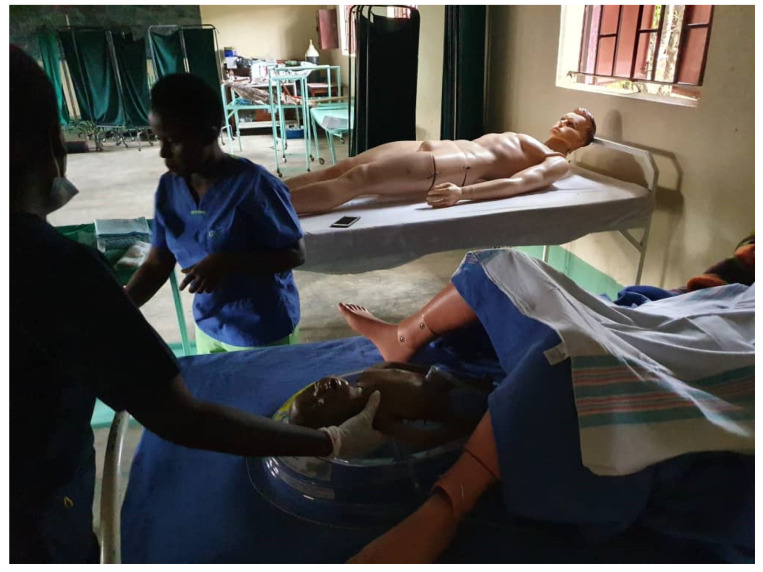
Demonstration of the BabySaver design at birth.

The weight of the developed design alone is 1850 g; the pieces of equipment for resuscitation weigh another 600 g. Figure 2 shows the final version of the BabySaver. Figure 3 shows its demonstration at birth with a model.

#### 3.2.1. The Tray

**Figure 4:**
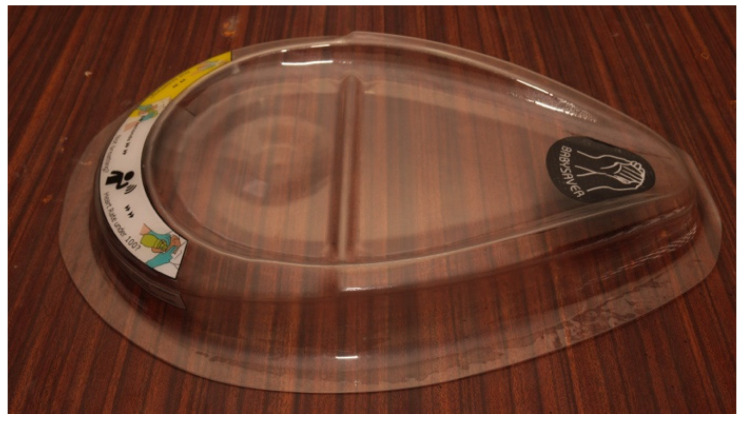
The tray.

The tray forms a clear plastic lid (Figure 4), that fits neatly into the base to form a toolbox. When the tray/lid is inverted, it forms a flat support platform. The platform provides a stable, clean, and smooth cradle to hold the neonate while the umbilical cord remains connected to the placenta at the time of birth. The weight of the tray is 950 g. The broad end is 455 mm wide, the longest length 685 mm and the depth 70 mm. It is 2 mm thick.

The tray has a groove to receive and stabilise the baby’s head. The groove is a few millimetres deep. The raised neck support slightly extends the baby’s neck, positions the head into the groove, and keeps the neck in a neutral position with ease.

**Figure 5:**
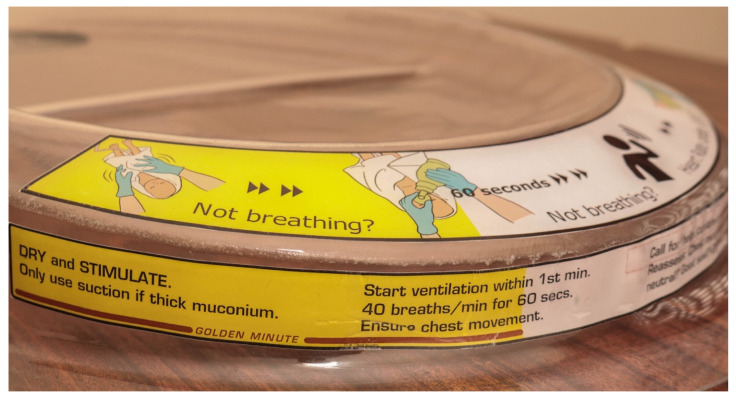
Pictorial instructions of helping babies breathe on the tray.

The tray has adhesive labels on its under surface which ensure that the top surface is completely smooth. The labels, visible through the plastic, carry icons and abbreviated instructions to act as a reminder for the skilled birth attendant (SBA) (Figure 5). These resuscitation instructions were adapted from the HBB programme [36], which is widely used in Uganda. It is easy to clean the tray.

#### 3.2.2. The Base

The base is a non slip white plastic compartment. It has space for all the essential pieces of equipment for resuscitation at birth recommended for HBB [31] and the supplies necessary for essential newborn care for every baby at birth.

The base is specifically in white to easily detect any stain and is suitable for use on the resuscitation table, a delivery bed, an operation table, or any other available surface.

**Figure 6:**
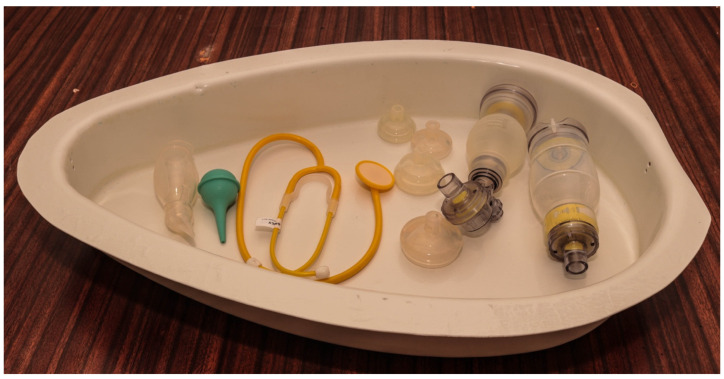
The base.

The weight of the base alone is 900 g. It is 103 mm deep with the widest broad end measuring 455 mm and its length is 685 mm. It is 3 mm thick, Figure 6.

## 4. Discussion

Providing neonatal resuscitation at the bedside with an intact umbilical cord is potentially a high benefit practice with major global benefits [9,10,11]. The development of a low-cost and sustainable platform is central to this process. We have described the design process for a device that enables neonatal resuscitation with an intact umbilical cord without taking the midwife away from the mother’s delivery bed [9,10,11]. This is the first device designed for use where there is a lone midwife on duty in the labour and delivery suite. This is a common occurrence in the developing world, where neonatal resuscitation at birth is usually provided by the attending midwife unlike settings with appropriate staffing where staff are solely responsible for the care of the newborn [27].

The BabySaver has the potential to be used in any other low-resource settings outside Uganda, and outside hospital settings by trained birth attendants. The midwives could have the BabySaver readily available with them for use in emergency and or unplanned births places such as home, the roadside, en route to the hospital, etc.

Every effort has been made to make the design user-friendly. The shape of the design follows nature; for us in Uganda, the egg gives life, the egg-shape of a BabySaver can remind us that its purpose is to enable the life of a baby. The design is more eco and user-friendly, simpler and easier to use than the designs, such as LifeStart trolley [12,13,14,15] and the Concord trolley [16,17,18,19]. The BabySaver is suitable as a basic resuscitation platform for use in high-resource settings. Midwives in the United Kingdom could have the BabySaver in the back of their cars for roadside births that require resuscitation.

The initial design was different from the final design. Although medical devices need to satisfy their intended purpose, considering the context for the device to operate efficiently is primary when designing medical devices for low resource settings [37]. We hence considered individual level socio-cultural factors, physical labour and delivery suite environments, health facility structures, and systems as the context that informed the final design. We encourage other designers of any related device to include a heating function to warm the baby during resuscitation, and any other specification according to local needs and resources.

The timer was removed from the final design based on the recommendation of the end-users. Various methods for time function are already in place during resuscitation. Midwives usually use the second hand on the wall clock in the labour suite, nurses’ watches, and mobile smartphones with a time function.

The instructions displayed on the design were in line with best practice resuscitation recommendations and the local guidelines for helping babies breathe (HBB) [4,31,38]. Though the design allows the practice of resuscitation before clamping the cord, it does not change the standards or steps of resuscitation [38].

Less than two hours of training are required before the BabySaver can be used in practice, which can easily be integrated into the HBB training curriculum [38,39].

The position of the device at the time of resuscitation is in between the mother’s legs. This compares favourably with the mother’s side position of the LifeStart trolley and Concord trolley [20]. This position provides an optimal position to allow placental transfusion by gravity to the depressed term neonates at birth.

The device is currently suitable for use on a resuscitation table, a delivery bed, an operation table, or any other available surface. However, we do not recommend its use on the mother’s chest/abdomen due to its inability to provide blood transfer by gravity. While the device can be introduced and used in the operating theatre, this is only recommended after ensuring sterility will be maintained. We propose sewing linen in a pocket fashion, following the shape of the BabySaver tray, sterilizing it, and having the tray dressed in sterile linen.

It took about two years to achieve the definitive design. This included a delay of about 8 months while seeking funds. We would expect designers of any related device to need less time, approximately 6 months if funding is secured.

In our design process, the outcome was a design that responded to the local needs of the users and the delivery environment [37]. We iteratively and collaboratively designed this device with women, health workers, and the public. Patient and public involvement (PPI) is increasingly considered an integral part of research and innovations [40,41]; the involvement of diverse groups of users throughout the design process increases the likelihood of a successful design [41].

The current prototype has undergone phase I/II usability clinical testing. There are plans to refine the device based on feedback from the usability testing before checking for its clinical effectiveness in community health facilities in Uganda. The clinical effectiveness data will inform the scale of the device in Uganda, across other low-resource countries, Islamic Development Bank member countries, and any other interested high-resources settings.

## 5. Conclusions

This is the first mobile resuscitation device developed to facilitate the resuscitation of newborns in between the mother’s legs with placental transfusion at birth. The name BabySaver implies a commitment to saving neonates at birth. Further studies will assess its feasibility, efficacy, safety, and acceptability in the delivery rooms in Uganda. An effectiveness trial will be conducted after the results of the feasibility study.

## Figures and Tables

**Table 1:** Situational analysis of resuscitation in labour and delivery suites in Uganda (2015).

S/No	Resuscitation Procedure in Delivery Rooms	Problems with the Current Method of Resuscitation at Birth	Mechanism of Action	Outcome
1	The baby is delivered by attending skilled birth attendant or student in the labour suite or operating theatre			
2	The baby’s cord is clamped, or tied immediately and cut by the skilled birth attendant or student	Lack of a system to perform delayed cord clamping	Leads to decreased blood volume, haemoglobin and iron stores	An effect on baby’s neurological development
3	The baby is dried and kept warm by the attending skilled birth attendant or student	Transfer of baby to a separate resuscitation station	Maternal-baby separation leading to fear of babies being swapped	Maternal distress
4	The baby is transferred to a resuscitaire/resuscitation station if it does not cry	Resuscitaire is not functional or broken	Resuscitation may be performed inadequately on available surfaces	Unnecessary death
5	The baby’s airways are checked for any secretions and cleared if needed by wiping with a cloth or suctioning	Lack of constant electricity in delivery rooms	Absence of a heating option for the baby on the resuscitaire	Risk of hypothermia during resuscitation on the resuscitaire
6	The baby’s back is rubbed 2−3 times, or feet slapped to stimulate breathing	Inconsistent or unavailable in-service training programme on helping babies breathe	Midwives not up to date with resuscitation guidelines or steps	Inability to follow guidelines for resuscitation
7	The baby is positioned in neutral position and ventilation initiated with bag and mask	Missing equipment for resuscitation	Inability to perform all the necessary steps of resuscitation	Unsuccessful resuscitation
8	If available, a mixture of oxygen and air is recommended for premature babies	Equipment is not in one place at time of resuscitation	Delays to initiate suctioning, bag and mask ventilation	Wasting of golden minute
9	If required after bag and mask ventilation, chest compressions are performed on the baby by a midwife			
10	If these are unsuccessful and drugs are available, drugs are administered by attending skilled birth attendant			
11	If resuscitation is successful, the baby is transferred to the neonatal unit			
12	The equipment used in the birth is taken for cleaning by the attending midwife	The equipment is cleaned in the same buckets as those for delivery		
13	The resuscitation equipment is maintained by the local engineering department			

**Table 2:** Design specification (the BabySaver wish list) in 2015.

S/No.	Requirement	Comments	Rank
1	The design must warm the baby to prevent hypothermia.	The heating should not harm the baby	1
2	The design must work with resuscitation protocols in place.	The systems may vary from hospital to hospital, but the tray should fit into standard practice in Mulago and Mbale hospitals	2
3	The design must be made of non-slip, cleanable materials.	The materials used must be able to be sterilised using current local cleaning practices	2
4	The design must provide pictorial instructions on how to deliver neonatal resuscitation.	This must agree with up-to-date standards of practice	3
5	The design must have storage capabilities for auxiliary equipment.		4
6	The design must be able to be moved safely.	This includes when in use	5
7	The design should be cheap to manufacture	Aim for manufacture price under $10	6
8	The design should have a light.	This should be independent from hospital utilities	7
9	The design should have a timer.		8
10	The design should be theft proof.	If possible, the design should discourage theft, or theft of parts.	9
11	The design should be able to be used on a variety of surfaces.		

**Table 3:** Equipment for resuscitation in the base of the BabySaver design.

Equipment and Supplies for the Care of Every Newborn at Birth in the BabySaver		
Essential equipment	Optional equipment	Optional Supplies
Suction device	NeoBeat newborn heart rate monitor	Surgical gloves
Neonatal Ambu Bag	Laryngoscope	Cord ties
Neonatal face mask size 1		Timer
Neonatal face mask size 0		Tetracycline eye ointment
Neonatal stethoscope		Vitamin K
		Intravenous fluids
		Adrenaline
		Oxytocin

**Table 4:** Risk assessment for the BabySaver design using a Failure mode and effects analysis (FMEA) form.

Key Process	Potential Failure Mode (FM)	Potential Failure Effects	SEV	Potential Causes	OCC	Current Controls	DET	RPN	Recommendations	Responsible Person
What is the process step or input?	In what ways can the process step or input fail?	What is the impact on the key output variables once it fails (customer or internal requirements)?	How severe is the effect on the customer?	What causes the key input to go wrong?	How often does cause or FM OCC occur?	What are the existing controls and procedures that prevent either the cause or the failure mode?	How well can you detect the cause or the failure mode?		What are the actions for reducing the occurrence of the cause, or improving detection?	Who is responsible for the recommended action?
Organise resuscitation equipment in the BabySaver	The bag and mask are non-functional	The baby receives inadequate ventilation	1	Defects in the pieces of equipment and limited user ability to detect early	1	All resuscitation equipment included in BabySaver should be checked before handing over to the hospital staff	1	4	User training	supplier/SAfRI/Laerdal Global
BabySaver is brought close to the delivery bed	BabySaver falls	Midwife is injured	2	BabySaver held with slippery hands and in a rush	2	There is usually a delivery trolley in the labour suite, on which the BabySaver can be placed and moved	1	4	The users are trained on handling the BabySaver unit in a real clinical setting	Skilled birth attendant
Explain BabySaver to the mother and attendant	Mother scared of the device	Mother refuses the BabySaver use	2	Deliveries occurring before the explanation. Many deliveries under 1 midwife	2	All women are admitted to labour at a central station	1	4	Explanation of the device at any opportunity a midwife is in contact with the mother in labour during admission, examinations, etc.	Skilled birth attendant
Place the tray in between mother’s legs	Tray slips	Mother and Baby are injured	2	A tray placed on an incompatible surface, no space in between legs. The tray is positioned wrongly with the broad end closet to the buttocks instead	2	Some delivery beds are long enough in the labour suite. For the surface material of the delivery beds, tests need to be performed to see how feasible this is.	1	4	User training on the positioning of the BabySaver, feasibility testing	Skilled birth attendant
Activates gel pack	The heat gel pack is not present	Baby’s temperature drops	1	Equipment not replaced after use	3	The tray will have a checklist of equipment that should be included	1	3	Equipment included will be detailed in the instructions	Manufacturer
	Heat pack ruptures	Baby is injured	2	Heat pack has weak point due to incorrect care or manufacturing flaw	1	Pack contents non-toxic, pack contents not hot enough to injure	1	2	Instructions on pack care (not having the plastic on a hot surface, wrapping with a cloth while boiling, etc.) included	Manufacturer
	Heat pack overheats	Baby is injured	3	The pack is placed directly on neonate skin instead of through cloth	1	The heat gel pack has been tested to ensure it does not reach harmful heat levels	1	3	Instructions to regularly check the infant for erythema included	Manufacturer
	Heat pack underheats	Baby’s temperature drops	1	Manufacturing error	1	Spare heat pack provided	2	2	Batch testing required	Manufacturer
Gently place the baby onto the tray	The baby is placed incorrectly	Resuscitation is incorrectly performed	1	Insufficient training	3	Neck support and pictorial instructions included	1	3	User training	Supplier/SAfRI/manufacturer
Position the baby in a neutral position	The baby is positioned incorrectly	Resuscitation is incorrectly performed	1	The neck support is too elevated, or the neck support groove is too shallow	3	Minimum elevation of the neck support included	1	3	User training	Manufacturer
Keep the baby warm	The baby loses heat on the tray	Baby develops hypothermia	1	The baby is not covered in enough clothes, cotton dry clothes. Wet clothes not changed	3	The baby is dried and changed into a second warm cotton cloth before starting resuscitation	1	3	User training	Skilled birth attendant
Resuscitation is performed	Instructions are unclear	Resuscitation is incorrectly performed	3	Insufficient user training	1	Instructions are standard internationally agreed guidelines, checked with experienced practitioners for content.	2	6	User training, instructions also checked with inexperienced users for clarity	Supplier
	Tray fails mechanically	Mother and baby are injured	2	Too much force on the tray; sharp edges can cause discomfort	1	Durable material that does not degrade, smooth surface edges	2	4	Product lifecycle and surfaces of the edges advised in instructions	Manufacturer
	Mother kicks the tray during use	Midwife is injured	2	Maternal distress	1	Instructions for the user	2	4	User training and explaining to the mother early about the use of the BabySaver	Skilled birth attendant
Withdraw the tray from between mother’s legs	The tray slips or is transferred to the ground	Midwife and mother injured	1	user oversight and fatigue, slippery gloves soiled in liquor and blood	3	The tray has a flat flap for firm handling	1	3	The users must be trained on proper handling of the tray with both hands where possible	Users/skilled birth attendants
Withdraw the equipment from the delivery field	Equipment is not replaced	Resuscitation is incorrectly performed	1	Replacement is not available, user oversight, pieces of equipment for resuscitation remain in mother’s clothes	3	The tray has a checklist of equipment that should be included	1	3	Equipment included will be detailed on the instructions, as well as care instructions for the auxiliary components	Manufacturer
Reprocess the BabySaver and equipment	BabySaver is not disinfected fully	Baby contracts infection	2	Cleaning protocol not followed	3	The product has no crevasses; is compatible with local cleaning products	2	12	User training	Supplier
	BabySaver is not disinfected immediately	Baby contracts infection	2	Cleaning protocol not followed	3	The product has no crevasses; is compatible with local cleaning products	2	12	User training	Supplier
	Baby’s pieces of equipment for resuscitation are mixed with maternal delivery pieces of equipment	Baby contracts infection	2	Cleaning protocol not followed	3	The product has no crevasses; is compatible with local cleaning products	2	12	User training	Supplier
The tray is returned to storage	Equipment is not replaced	Resuscitation is incorrectly performed	1	Replacement s not available, user oversight	3	The tray has a checklist of equipment that should be included	1	3	Equipment included will be detailed on the instructions, as well as care instructions for the auxiliary components	Manufacturer

SEV-Severity. OCC-Occurrence. DET-Detectability. RPN-Risk Prioritisation Number. FMEA-Failure Mode and Effects Analysis.

## Data Availability

The paper did not report any data, but unpublished technical files of the design are available upon request from the corresponding author.
